# Cerebral sparganosis presenting with atypical postcontrast magnetic resonance imaging findings: a case report and literature review

**DOI:** 10.1186/s12879-019-4396-2

**Published:** 2019-08-27

**Authors:** Yueli Zhu, Lingqi Ye, Xiansan Ding, Jimin Wu, Yanxing Chen

**Affiliations:** Department of Neurology, the Second Affiliated Hospital, School of Medicine, Zhejiang University, Hangzhou, China

**Keywords:** Cerebral sparganosis, Tunnel sign, ELISA, Praziquantel

## Abstract

**Background:**

Sparganosis, a rare and severe parasitic infection caused by the larvae of *Spirometra* species or simply sparganum, generally involves subcutaneous tissue or muscle. But occasionally, sparganum can also invade the human brain, resulting in cerebral sparganosis.

**Case presentation:**

A 33-year-old woman presented with a 10-day history of headache. Postcontrast magnetic resonance imaging (MRI) revealed an irregular lesion with enhancement and the tunnel-shaped focus extending to the contralateral hemiphere. Cerebrospinal fluid (CSF) analysis disclosed pleocytosis (166 cells/μL) and an elevated protein concentration (0.742 g/L). Enzyme-linked immunosorbent assay (ELISA) revealed positive sparganum-specific antibody in both blood and CSF. Finally, the diagnosis of cerebral sparganosis was comfirmed. She received praziquantel treatment and got a favorable outcome during six-month follow-up.

**Conclusions:**

Irregular enhancement and the tunnel sign that extends to the contralateral hemisphere on postconstrast MRI are unusual presentations of cerebral sparganosis. ELISA for sparganum-specific antibody can help confirm the diagnosis. Although surgery is the preferred treatment for cerebral sparganosis, praziquantel might also achieve satisfying outcomes.

## Background

Sparganosis is a severe and uncommon parasitic disease caused by infestation of sparganum [1, 2]. The sparganum commonly invades the subcutaneous tissue and muscle, and the invasion to the brain is rare [3, 4]. Wild dogs and cats, whose intestines are parasitized by adult *Spirometra* species, spread eggs produced by *Spirometra* species via defecating. These eggs grow into coracidia in water. Then coracidia are ingested by copepods (first intermediate host) and grow into procercoid larvae. These larvae are infective to frogs, snakes, birds, and mammals in which procercoids mature into plerocercoid larvae [5, 6]. There are three routes of infestation with sparganum in human, including drinking water contaminated with infected copepods, eating raw or undercooked frog, snake, chicken, or pork meat, and using the flesh of an infected intermediate host as a poultice to open wounds [7, 8]. The common symptoms of cerebral sparganosis are seizures, headache, hemiparesis, and sensory disturbance, depending on the sites of lesions [9, 10]. When the clinical manifestations are nonspecific, characteristic signs on brain computed tomography (CT) and magnetic resonance imaging (MRI) have been reported to be useful to make an accurate diagnosis, which include tunnel sign on ipsilateral hemisphere and ring-like enhancement [11]. Surgery is recommended for this disease [9].

Here, we present a rare case of cerebral sparganosis with unusual MRI findings that remained a diagnostic challenge for neurologists. The patient achieved a good outcome during follow-up with the treatment of praziquantel without surgery. We also reviewed the literature about previous cases of this disease with complete postcontrast MRI.

## Case presentation

**Fig. 1:**
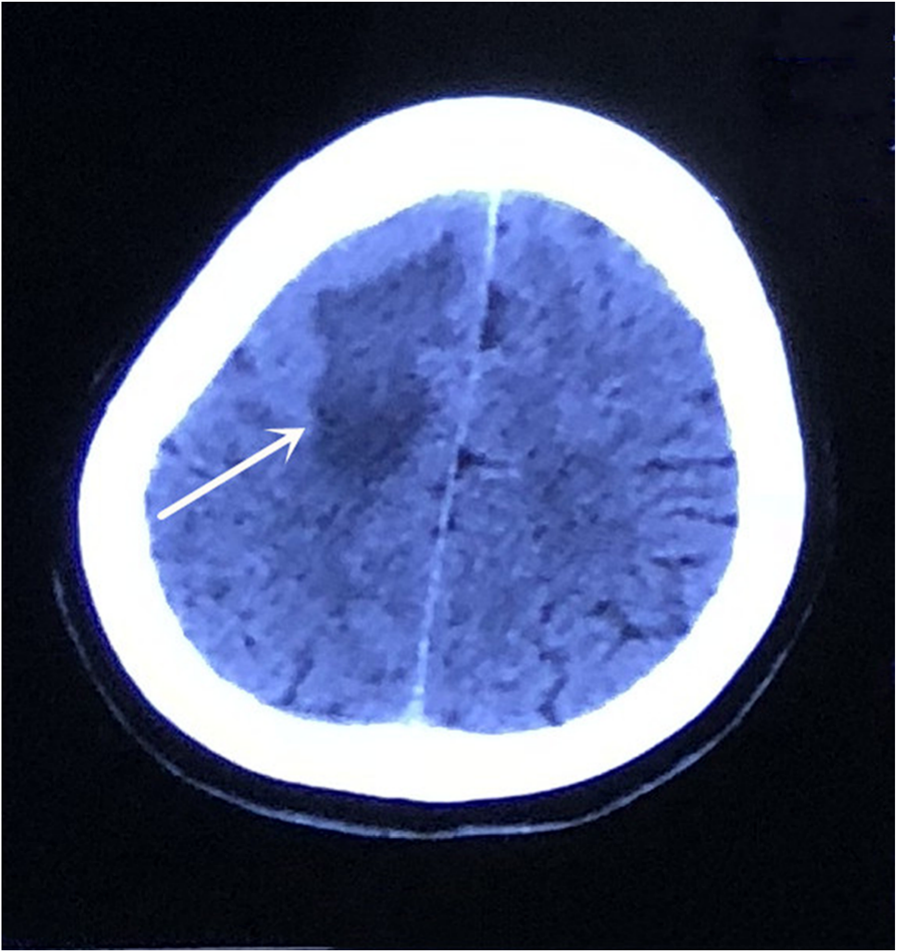
Unenhanced CT was performed at the onset of headache and showed an area of hypodensity in the right frontal lobe

**Fig. 2:**
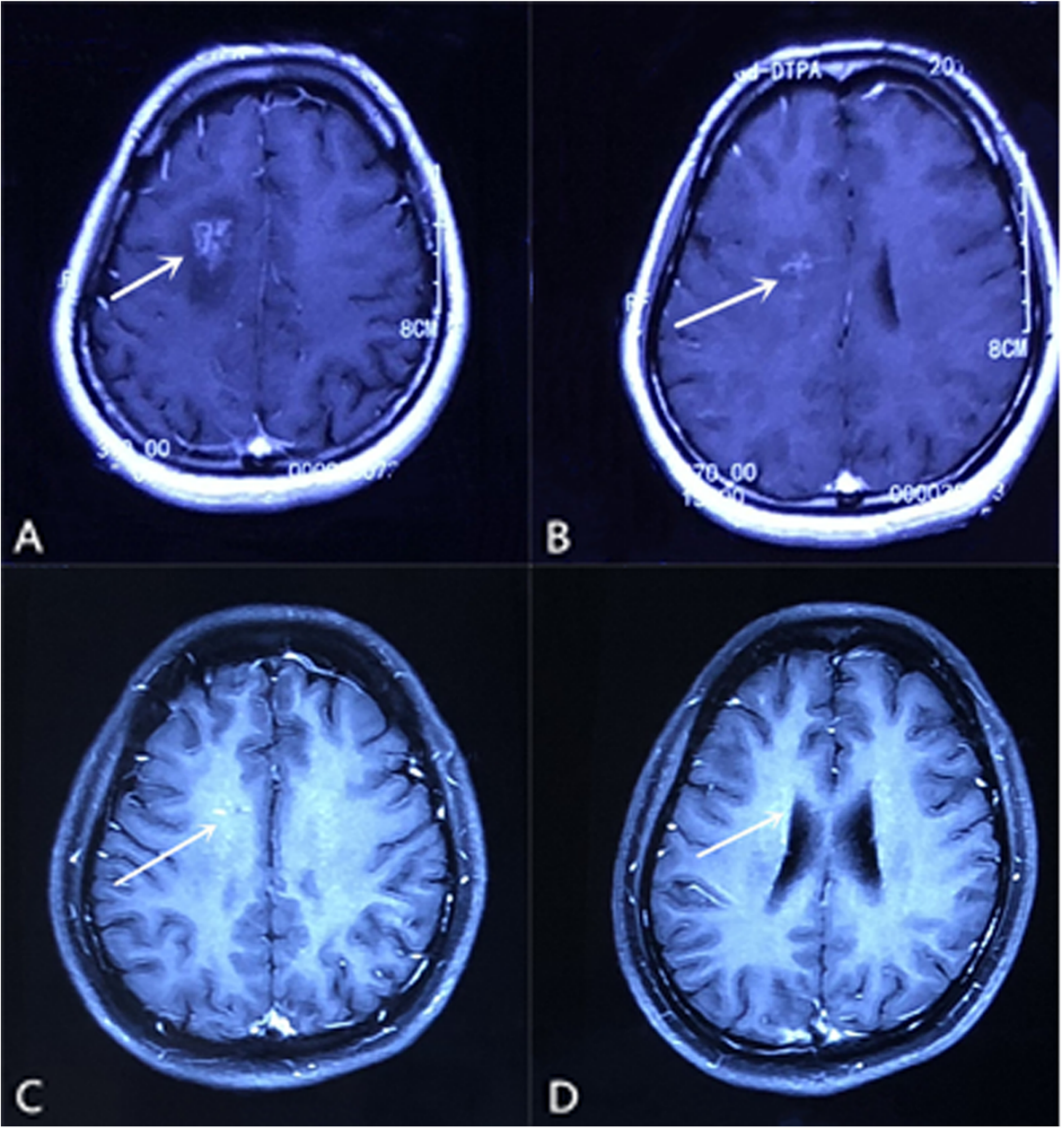
Cranial postcontrast MRI obtained at the onset of headache revealed an irregular enhancement lesion with perifocal edema in the right frontal lobe (**a**) and tunnel sign involving the bilateral brain (**b**). Postcontrast MRI performed after the second two-day praziquantel treatment, the irregular enhancement lesion was obviously reduced (**c**) and tunnel sign was almost invisible (**d**)

A 33-year-old woman, a nurse, was referred to our hospital due to headache for 10 days. She had a brain CT scan at a local hospital on June 5, 2018, which revealed a hypodensity lesion in the right frontal lobe (Fig. 1). Brain MRI was also performed on the same day. An irregular lesion, hypointense on T1-weighted imaging (T1WI) and hyperintense on T2-weighted imaging (T2WI), was observed. Enhanced scans showed irregular enhancement with perifocal edema (Fig. 2a). Besides, tunnel-shaped focus was observed, involving the bilateral brain (Fig. 2b). The patient was suspected to have demyelinating pseudotumor and was treated with 10 mg dexamethasone for 5 days. After the treatment, her headache relieved.

When she was admitted to our hospital on June 12, 2018, the general physical examination and neurological examination revealed no abnormality. Routine haematological and biochemical investigations were normal. The brain MRI performed in our hospital on June 15 revealed similar findings to that performed on June 6. Lumbar puncture revealed normal cranial pressure. CSF analysis disclosed 166 cells/μL and protein concentration of 0.742 g/L. The levels of glucose and chloride were normal. Bacterial and fungal cultures were negative. A postcontrast MRI was ordered. The tunnel-shaped lesion involving the contralateral hemisphere caused our attention, which was clearly seen in the body of corpus callosum. Based on her CSF and imaging findings, parasitic infection was suspected. ELISA showed positive anti-sparganum antibody in both blood and CSF.

The patient was thus diagnosed as cerebral sparganosis. However, it remained unclear how she got infected by this rare parasite. She denied having drunk contaminated water, eaten raw or undercooked frog, snake, chicken, or pork meat, or used the flesh of them as a poultice to open wounds. The patient refused to have the surgery. She received praziquantel 25 mg/kg/dose 3 times daily for 2 days. Lumbar puncture and brain MRI were performed again to evaluate the treatment effects 1 week later. CSF analysis showed 120 cells/μL and protein concentration of 0.264 g/L, which was better than before. MRI showed that the lesion size was markedly reduced. The two-day course of praziquantel treatment was repeated, and she was then discharged with no signs or symptoms. A follow-up brain MRI on July 25 showed that the punctate enhancement was further reduced and the tunnel sign almost disappeared, indicating significant therapeutic effect of the treatment (Fig. 2c and d). No headache relapse or other neurological deficits were observed during six-month follow-up.

## Discussion and conclusions

We report here a peculiar case of cerebral sparganosis with uncommon brain MRI findings. The patient got a favorable prognosis with praziquantel treatment.

The cycle and infective routes of sparganum are well documented. Because of the big size of sparganum (approximately 50 mm in length and 1 mm in width), cerebral sparganosis is most likely caused by drinking water contaminated by copepod that contains tiny procercoid stage of larva [10, 12]. When a patient gets infected, the larvae enter the abdominal cavity by passing through the alimentary canal. Then they further migrate into the diaphragm and mediastinum to reach the neck. At last, they pass through the foramen magnum and enter the brain [6]. The most common symptoms include seizures, headache, hemiparesis and sensory disturbance, depending on the sites of lesions [9, 10]. The course of this disease sometimes is acute, mimicking the forms of subarachnoid hemorrhage or encephalitis. In most situations, it has a long duration varying from months to years, resembling the course of a granuloma [13]. In our case, the patient manifested with acute onset of headache. Eosinophilia occasionally exists in cerebral sparganosis [10], but it was not found in our patient. Because of its rarity and lack of characteristic manifestations, cerebral sparganosis is easily misdiagnosed.

It has been reported that the characteristics of cerebral sparganosis on CT include the presence of unilateral involvement, low-density lesions in the white matter with ipsilateral ventricular dilatation and localized cortical atrophy, nodular or irregular enhancement with spotty calcification, and changes in the location of lesions [14]. These findings indicate long-term inflammation with active granuloma and irreversible brain damage on account of worm migration and histotoxic effects caused by proteases secreted from the worm [15]. In the present case, only an apparent low-density lesion without ventricular dilatation or cortical atrophy was observed on brain CT. It may be related to the short time from onset to diagnosis, as it is proposed that atrophic change is the result of chronic pathological course [4]. Spotty calcification was not observed either on brain CT of the current case.

Postcontrast MRI is supposed to be superior to CT scan for the diagnosis of most cerebral parasitic diseases [11, 15]. To our knowledge, only 15 reports searched from Pubmed presented clear postcontrast MR images (Table 1). Most cases were reported in Japan, China and Korea. We summarized the characteristics of all the 16 cases, including the current one, in Table 1. Eight patients had suspicious infection history. The main infectious source was eating raw or undercooked frog, snake, chicken, or pork meat. The remaining eight patients (including the present case reported here) could not recall any of these possible infectious sources. Thus, it remains elusive how these patients became infected. Literature review indicates that seizure is the most common clinical manifestation, followed by headache, sensory disturbance, hemiparesis, dysarthria and hemianopia. The major sympotoms are similar to those reported by Kim et al. from South Korea [31]. The course of the disease varied from 1 month to 10 years. It was reported that ring-like enhancement is the common finding and the tunnel sign is the most characteristic finding on postcontrast MRI [11, 32]. The ring-like enhancement represents an inflammatory granuloma [17]. And the tunnel sign is formed by reactive inflammatory tissue or granuloma enwrapping the worm, suggesting the movement of the larva [11, 33]. Nearly all cases shown in Table 1 have the ring-like / bead-like /nodular enhancing lesions except for our case, which showed an irregular shaped enhancement. The tunnel sign indicates the migration of live worm, which always occurs on the same hemisphere [11, 33, 34]. However, in the current case, an apparent tunnel sign in the body of corpus callosum, extending to the contralateral hemisphere, was observed on brain MRI, which has never been reported before. It is suggested that the detection of sparganum-specific antibody in the CSF and blood samples of a suspected patient by ELISA can be highly sensitive and specific [35]. ELISA was performed in 9 cases and sparganum-specific antibody was identified in the blood samples and/or CSF samples of 8 patients (including the current case). Thus, ELISA test is useful for the correct diagnosis of cerebral sparganosis in addition to imaging findings and relevant history.

**Table 1:** Clinical characteristics of 16 patients with cerebral sparganosis

Age (yrs) /Sex/Ref	Country	Mode of infection	ClinicalManifestation	Enhanced MRI(appearance)	ELISA	Treatment	Outcome/Follow-up time
29/F/ [16]	India	Uncertain	Seizure/7 mo	Nodular	NP	Craniotomy	Improved/9 mo
29/M/ [17]	Spain	Water	Seizure/4 y	Ring-like	NP	Craniotomy	Improved /1 y
71/M/ [18]	Japan	Uncertain	Dysarthria	Bead-like	BLD +	Craniotomy	Improved /2 y
18/F/ [19]	China	Food	Headache and seizure/1 mo	Bead-like	BLD + CSF +	Abendazole	Improved /1 y
38/M/ [20]	Japan	Uncertain	Headache, fever and cerebellar ataxia/2 y	Ring-like	BLD + CSF +	NR	NR
39/M/ [21]	Switzerland	Uncertain	Seizure	Ring-like	BLD + CSF +	Praziquantel	Improved/11 mo
15/F/ [22]	China	Food	Numbness/1 y seizure/6 mo	Ring-like	BLD +	Craniotomy	Improved /2 y
24/M/ [23]	Korea	Uncertain	Headache/1 mo	Bead-like	NP	Craniotomy	NR
62/M/ [24]	Japan	Food	Seizure and hemiparesis/10y	Ring-like	BLD - CSF -	Craniotomy	Improved /5y
64/M/ [25]	Japan	Food	Seizure and weakness	Ring-like	BLD + CSF +	Craniotomy	NR
48/M/ [26]	Korea	Food	Weakness and hemianopia	Nodular	BLD + CSF +	Craniotomy	Improved
80/M/ [27]	Japan	Water	Numbness and weakness	Nodular	NP	Craniotomy	Improved
21/M/ [28]	America	Uncertain	Confusion/18 mo and seizure once	Ring-like	NP	Craniotomy	Improved
25/M/ [29]	Germany	Uncertain	Headache and seizure	Ring-like	NP	Craniotomy	Improved
6/M/ [30]	China	Water	Seizure/2 y	Bead-like	NP	Craniotomy	Improved/6mo
33/F/PR	China	Uncertain	Headache/10 d	Irregular	BLD + CSF +	Praziquantel	Improved/6mo

*F* Female, *M* Male, *Yrs* Years, *NP* Not performed, *NR* Not reported, *PR* Present case, *ELISA* Enzyme-linked immunosorbent assay, + Positive, − Negative, *BLD* Blood, *CSF* Cerebrospinal fluid

The differential diagnosis of cerebral sparganosis should include chronic cerebral ischemia, brain tumors and inflammatory granulomas of various causes, such as mycosis, tuberculosis or other parasitic infections [7, 11, 14]. Chronic cerebral ischemia is relatively easy to be distinguished because of the lack of ring-like enhancement or tunnel sign on MRI. Brain tumors can mimic cerebral sparganosis on CT and MRI, but mass effect compressing the lateral ventricle can often be found while sparganosis shows adjacent ventricular dilation [11]. Inflammatory granulomas may present as a round enhancement with or without surrounding edema, but brain tissue atrophy or dilation of the adjacent ventricle generally does not exist [14]. When the lesions do not have response to the treatment, other diagnosis should be considered [16]. On postcontrast MRI, tumors and inflammatory granulomas do not usually have the tunnel sign as cerebral sparganosis. It is worth noting that CSF and blood can occasionally offer specific clues with elevated eosinophils [36], but were normal in our case. The lesson for us is that irregular enhancement and tunnel sign involving the contralateral hemisphere sometimes can appear in cerebral sparganosis. ELISA can be helpful in the accurate diagnosis and should be performed when special neuroimaging results are observed.

The preferred treatment for cerebral sparganosis is surgical removal. Oral medication, including praziquantel, is deemed unable to kill a live worm or achieve beneficial clinical effects [7, 9, 30]. However, recent studies indicated that high-dose praziquantel therapy can be effective in patients with cerebral sparganosis [32, 37, 38]. Of the 15 cases we summarized above, only two patients received oral medication, abendazole and praziquantel, respectively (Table 1). Both of them had good outcomes. Our patient was treated with conventional-dose praziquantel because of her refusal of surgical removal. Fortunately, she made a good recovery and no neurological relapse was observed during six-month follow-up. Repeated brain MRI also revealed much smaller lesions. Hence, we believe that drug treatment should be considered in cases where surgery is unacceptable or inoperable.

In conclusion, we report a case with atypical MRI findings of cerebral sparganosis. To the best of our knowledge, the tunnel sign which extends to the contralateral hemisphere on MRI has never been reported. Once cerebral sparganosis is suspected, ELISA for sparganum-specific antibody should be applied. Although surgical removal is generally regarded as the best treatment for cerebral sparganosis, drug treatment, such as with praziquantel, sometimes can achieve satisfying outcomes as well.

## Data Availability

The datasets used and/or analyzed during the current study are available from the corresponding author on reasonable request.

## Abbreviations

CSF: Cerebrospinal fluid
CT: Computed tomography
ELISA: Enzyme-linked immunosorbent assay
MRI: Magnetic resonance imaging
T1WI: T1-weighted imaging
T2WI: T2-weighted imaging
